# COVID-19 Vaccine Acceptance in a Sample From the United Arab Emirates General Adult Population: A Cross-Sectional Survey, 2020

**DOI:** 10.3389/fpubh.2021.614499

**Published:** 2021-07-26

**Authors:** Abdulaziz Hussain Albahri, Shahad Ahmed Alnaqbi, Asma Obaid Alshaali, Shatha Ahmed Alnaqbi, Shaikha Mohammad Shahdoor

**Affiliations:** Primary Healthcare Services Sector, Dubai Health Authority, Dubai, United Arab Emirates

**Keywords:** COVID-19, SARS-CoV-2, vaccine, vaccination, UAE, public perception, vaccine hesitancy, vaccine acceptance

## Abstract

**Introduction:** The COVID-19 pandemic has placed a tremendous stress on economies and healthcare systems worldwide. Having a vaccine is one of the promising solutions. However, vaccination hesitancy is becoming a recognized future challenge. This study aims to evaluate the current vaccine hesitancy in a segment of the United Arab Emirates (UAE) general public and its associated factors.

**Methods:** This was an online cross-sectional survey that took place from the 14^th^ to the 19^th^ of September 2020 across the UAE. The questionnaire asked the participants about their willingness to receive the COVID-19 vaccine in the future. Multivariable logistic regression analysis was used to assess the association between vaccination willingness and the participants' sociodemographic factors, experiences and beliefs regarding COVID-19, and previous influenza vaccine uptake.

**Results:** There was a total of 2,705 participants; 72.5% were females, and 69.8% were Emirati nationals. A total of 1,627 (60.1%) participants were willing to take the COVID-19 vaccine in the future. There were statistically significant associations between the following factors and vaccine acceptance: male gender, non-Emiratis, younger age group, residents of Sharjah and the Northern Emirates, having lesser educational attainment, perceived increased personal or public risk of contracting the disease [aOR = 1.71, 95% CI (1.35–2.17), *p* < 0.0001; aOR = 1.84, 95% CI (1.44–2.36), *p* < 0.0001, respectively], and increased perception of serious outcome from the disease. Conversely, vaccine hesitancy was associated with unemployment, not receiving the influenza vaccine within the past 2 years [aOR = 0.36, 95% CI (0.30–0.44), *p* < 0.0001], not believing in the seriousness of the COVID-19 situation or the vaccine's ability to control the pandemic, and not believing that the public authorities are handling the pandemic adequately. Having contracted the disease or knowing someone who has did not show a statistically significant association with vaccine acceptance. Vaccine safety, side effects, and the belief that one needs to develop immunity naturally were the top reasons for vaccination hesitancy.

**Conclusion:** Given the level of vaccine hesitancy in the study population, this needs to be evaluated in a more representative sample of the whole population. If confirmed, this would signify the need for coordinated local and international initiatives to combat vaccine misinformation and reassure the public regarding vaccine safety and efficacy.

## Introduction

The novel coronavirus disease-2019 (COVID-19) was first recognized in Wuhan, China, in 2019 and continues to challenge healthcare systems globally (1). It has caused considerable morbidity and mortality, with over 34 million infections worldwide by October 2020 and in excess of one million deaths (2). This respiratory beta coronavirus causes symptoms ranging from fever, cough, and shortness of breath, to non-respiratory symptoms such as loss of taste and smell, myalgia, and gastrointestinal symptoms (1). The scientific community and the pharmaceutical companies have raced to develop an effective vaccine against the severe acute respiratory syndrome coronavirus-2 (SARS-CoV-2), the causative agent of the disease. Various types of vaccines are being tested worldwide, and many of them are in phase 3 trials. These range from traditional inactivated virus vaccines such as the one developed by the Chinese Sinopharm company, to more novel platforms such as mRNA-based vaccines developed by Moderna and Pfizer-BioNTech companies, and adenovirus-based vaccines such as the two dose Oxford-AstraZeneca and the single dose Johnson & Johnson's Janssen vaccines (3, 4). In the UAE, the inactivated COVID-19 vaccine developed by the Chinese Sinopharm pharmaceutical company is undergoing a phase 3 clinical trial since the middle of July 2020 (4, 5). Having an effective vaccine at the earliest opportunity is of paramount importance to control the pandemic. At the same time, the safety of the individuals should not be compromised at any stage, whether during trials or at the post-marketing stage (6).

Vaccines, in general, have suffered from public hesitancy on many occasions, most recently leading to measles outbreaks (7). Reasons for hesitancy range from safety concerns to conspiracy theories to the need to develop immunity through natural infection (7, 8). A growing body of evidence is building that shows COVID-19 vaccination is not an exception (9, 10). Studies conducted in the USA, Europe, and elsewhere have shown a significant degree of hesitancy amongst the public to take the COVID-19 vaccine in the future for similar reasons to the ones stated above (10–13). Therefore, developing enough herd immunity to break the chain of the infection spread might prove to be a challenge. Some factors, such as being male, of higher educational attainment, and perceiving the risk of contracting COVID-19 as high, have been associated with greater willingness to take the vaccine (11–13). On the other hand, not receiving the influenza vaccine in the past and believing in anti-vaccine ideologies have been associated with greater hesitancy to uptake the vaccine (11–13). Most of these studies have been conducted in Western countries that have a different cultural and educational background to the Middle Eastern and Arab countries. In fact, vaccination hesitancy in general is a very poorly studied subject in the UAE and the Gulf region with very limited relevant data in the scientific literature. As a result, this study aims to explore COVID-19 vaccination willingness in a segment of the UAE general adult population and shed some light on the potential reasons for vaccination hesitancy in order to guide the future vaccination campaign locally and internationally.

## Methods

### Study Setting, Population and Sampling

**Figure 1:**
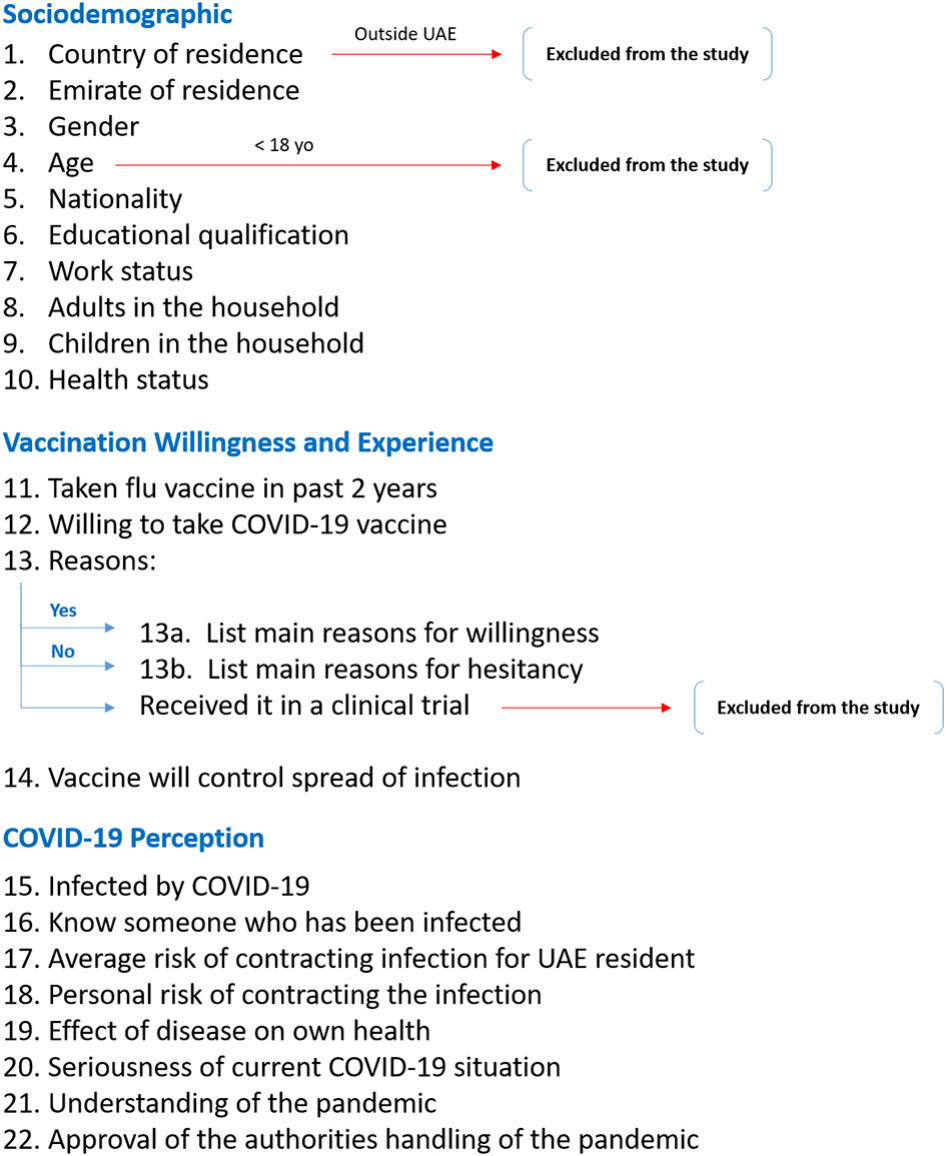
Flow diagram of the study questionnaire. This diagram illustrates the breakdown of the questionnaire structure and the exclusion points for certain participants regarding their country of residence, age, and willingness to take the vaccine.

This study was conducted over a six-day period from the 14^th^ to the 19^th^ of September 2020. Adults aged 18 years and older who were residing in the UAE at the time of the study were considered for the inclusion criteria. Any participant who received the COVID-19 vaccine as part of a clinical trial was excluded from the study, as illustrated in Figure 1.

The study sample size was calculated using the Epi Info v7.2.4 software (available at the CDC website: www.cdc.gov/epiinfo) based on a UAE population size of approximately 10 million, 95% confidence, and 2% margin of error. The sample size was estimated to be 2400. Sampling was performed using a snowballing technique mainly due to the pandemic situation and limitation to the human contact imposed by the social distancing rules; additionally, due to the lack of available representative database in UAE to select the representative sample from and the limitation in time to create such a database requiring swift responses and answers as the pandemic unfolds, this sampling technique was used. The investigators distributed the online questionnaire to their adult UAE resident contacts, and upon completion, they were asked to further forward it to their adult contacts residing in the UAE. The recruited participants received an explanatory page about the study and a consent sheet. Once consented, they were able to participate in the study. The questionnaire was mainly distributed through the WhatsApp messaging platform.

### Questionnaire Design

This online-based questionnaire was constructed based on previously published questionnaires investigating public hesitancy toward H1N1 vaccination, since at the time it was designed around late July 2020, very limited relevant COVID-19 acceptance questionnaires were available in the scientific literature (14–16). There were three categories of questions: sociodemographic factors, attitude toward COVID-19 vaccine and related perception, and personal attitude and perception toward COVID-19 and the pandemic. In the sociodemographic category, the participants were asked for their age, gender, nationality, emirate of residence, work status, educational level, number of adults in the household, presence of children in the household, and perceived own health status. This was followed by questions regarding whether they received the influenza vaccine within the past 2 years, whether they believe that the COVID-19 vaccine would help in curbing the spread of infection and controlling the pandemic, and lastly, if a COVID-19 vaccine becomes available to the public and proven to be effective, whether they would be willing to take it. Depending on the answer to the latter question, the participant was taken to a separate page and asked to select all his/her reasons for wanting or not wanting to receive the vaccine. Participants' experience with COVID-19 was explored via questions asking whether they had already contracted the infection, if they knew someone who had contracted the infection, perceived UAE public risk of contracting the infection, perceived own risk of contracting the infection, perceived possible health outcome if personally contracted the infection, perceived seriousness of the COVID-19 situation in general, whether they feel they are able to understand the pandemic and the COVID-19 situation, and whether they feel that the authorities are handling the situation appropriately. A copy of the questionnaire is provided in the Supplementary Material and Figure 1 outlines the structure of the questionnaire and the related exclusion criteria.

The questionnaire was translated into Arabic and back-translated, piloted on 30 individuals, and further modified in order to provide further clarity, suitability to the local setting, and consistency with the English version. Direct translation of the questions and answers from English into Arabic delivered mixed messages in the COVID-19 experience section, accordingly the questions and answers modified to ensure consistency in message delivery for both versions upon direct feedback from the pilot participants. Content and face validities were examined by experts in the field. A sample of both the English and the Arabic versions of the questionnaire are provided in the Supplementary Material. The data from the pilot study were excluded from the final analysis.

### Statistical Analysis

Statistical analysis was carried out using GraphPad Prism v8.4.3. The answers to the questions and the reasons for vaccine willingness and hesitancy were presented as counts and percentages. Multivariable logistic regression analysis was used to evaluate the association between vaccine acceptance and the participants' sociodemographic factors, experiences and beliefs regarding COVID-19, and previous influenza vaccine uptake. The logistic regression analysis was adjusted for the sociodemographic variables that had a univariate χ^2^ test with a *p*-value of <0.25, and has been evaluated for collinearity. Results of the logistic regression analysis were presented as adjusted odds ratios with *p*-value <0.05 considered as statistically significant.

### Ethical Approval

This study was approved by the Dubai Scientific Research Ethics Committee at the Dubai Health Authority. Participation in the study was voluntary, and all information was kept anonymous and confidential. An online consent was obtained prior to participation in the study.

## Results

### Sociodemographic Data

There was a total of 2,705 participants, 1,960 (72.5%) of whom were females. Most of them were from the younger age groups, with 63.0% aged 25–44 years and 194 participants (7.2%) aged 55 years and older. Over two-thirds of the participants were Emiratis (69.8%). Residents of Dubai constituted 55.7% of participants, followed by Sharjah (19.9%), Abu Dhabi (12.3%), and the Northern Emirates (12.0%). More than half of the participants reported having an undergraduate degree (58.6%); similarly, 58.3% stated being employed, while 32.7% were unemployed or retired. Four-fifths of the participants reported having more than two adults in the household (82.3%); likewise, 80.1% reported having at least one child in the household. Only 55 (2.0%) participants perceived their health as being poor-very poor. The sociodemographic data are summarized in Table 1.

**Table 1:** Sociodemographic characteristics, COVID-19 and vaccination perceptions of study participants and their associations with the willingness to take COVID-19 vaccine in the future (*n* = 2,705).

**Variables**	**Total**		**Willingness to be vaccinated**				**Univariate analysis**				**Multivariable logistic regression**	
			**Willing**, ***n*** **=** **1,627**		**Not willing**, ***n*** **=** **1,078**							
			**(60.1%; 58.25–61.95)**		**(39.9%; 38.05–41.75)**							
	***N* (%)**	**[95% CI]**	***N* (%)^**a**^**	**[95% CI]**	***N* (%)^**a**^**	**[95% CI]**	**χ2**	***P*-value**	**COR [95% CI]**	***P*-value**	**aOR [95% CI]**	***P*-value**
**Demographics**												
**Gender**^**#**^												
Female	1,960 (72.5)	[70.82–74.18]	1,070 (54.6)	[52.4–56.8]	890 (45.4)	[43.2–47.6]	91.65	^****^	Ref.		Ref.	
Male	745 (27.5)	[25.82–29.18]	557 (74.8)	[71.68–77.92]	188 (25.2)	[22.08–28.32]			2.46 (2.04–2.97)	^****^	2.37 (1.92–2.93)	^****^
**Age**^**#**^												
18–24	304 (11.2)	[10.01–12.39]	226 (74.3)	[69.39–79.21]	78 (25.7)	[20.79–30.61]	45.65	^****^	Ref.		Ref.	
25–44	1,704 (63.0)	[61.18–64.82]	1,038 (60.9)	[58.58–63.22]	666 (39.1)	[36.78–41.42]			0.54 (0.41–0.71)	^****^	0.56 (0.38–0.82)	^**^
45–54	503 (18.6)	[17.13–20.07]	267 (53.1)	[48.74–57.46]	236 (46.9)	[42.54–51.26]			0.39 (0.29–0.53)	^****^	0.39 (0.25–0.59)	^****^
55+	194 (7.2)	[6.23–8.17]	96 (49.5)	[42.46–56.54]	98 (50.5)	[43.46–57.54]			0.34 (0.23–0.50)	^****^	0.32 (0.19–0.51)	^****^
**Nationality**^**#**^												
Emirati	1,887 (69.8)	[68.07–71.53]	1,095 (58.0)	[55.77–60.23]	792 (42.0)	[39.77–44.23]	11.69	^***^	Ref.		Ref.	
Other	818 (30.2)	[28.47–31.93]	532 (65.0)	[61.73–68.27]	286 (35.0)	[31.73–38.27]			1.35 (1.14–1.60)	^***^	1.45 (1.20–1.76)	^***^
**Current emirate of residence**^**#**^												
Dubai	1,508 (55.7)	[53.83–57.57]	845 (56.0)	[53.49–58.51]	663 (44.0)	[41.49–46.51]	35.39	^****^	Ref.		Ref.	
Sharjah	539 (19.9)	[18.4–21.4]	337 (62.5)	[58.41–66.59]	202 (37.5)	[33.41–41.59]			1.31 (1.10–1.60)	^**^	1.26 (1.02–1.56)	^*^
Abu Dhabi	334 (12.3)	[11.06–13.54]	208 (62.3)	[57.1–67.5]	126 (37.7)	[32.5–42.9]			1.30 (1.02–1.65)	^*^	1.14 (0.88–1.47)	0.33
Northern Emirates	324 (12.0)	[10.78–13.22]	237 (73.1)	[68.27–77.93]	87 (26.9)	[22.07–31.73]			2.14 (1.64–2.80)	^****^	1.88 (1.43–2.49)	^****^
**Level of education**^**#**^												
Postgraduate	581 (21.5)	[19.95–23.05]	326 (56.1)	[52.06–60.14]	255 (43.9)	[39.86–47.94]	36.24	^****^	Ref.		Ref.	
Undergraduate	1,585 (58.6)	[56.74–60.46]	916 (57.8)	[55.37–60.23]	669 (42.2)	[39.77–44.63]			1.07 (0.88–1.30)	0.48	1.27 (1.03–1.56)	^*^
High school and less	539 (19.9)	[18.4–21.4]	385 (71.4)	[67.59–75.21]	154 (28.6)	[24.79–32.41]			1.96 (1.53–2.51)	^****^	2.16 (1.65–2.85)	^****^
**Work status**^**#**^												
Employed	1,576 (58.3)	[56.44–60.16]	997 (63.3)	[60.92–65.68]	579 (36.7)	[34.32–39.08]	46.51	^****^	Ref.		Ref.	
Unemployed (including retired)	885 (32.7)	[30.93–34.47]	456 (51.5)	[48.21–54.79]	429 (48.5)	[45.21–51.79]			0.62 (0.52–0.73)	^****^	0.72 (0.60–0.88)	^***^
Student	244 (9.0)	[7.92–10.08]	174 (71.3)	[65.62–76.98]	70 (28.7)	[23.02–34.38]			1.44 (1.07–1.94)	^*^	0.89 (0.58–1.37)	0.60
**Number of adults in the household**^**#**^												
One- two	479 (17.7)	[16.26–19.14]	307 (64.1)	[59.8–68.4]	172 (35.9)	[31.6–40.2]	3.78	0.052	Ref.		Ref.	
More than two	2,226 (82.3)	[80.86–83.74]	1,320 (59.3)	[57.26–61.34]	906 (40.7)	[38.66–42.74]			0.82 (0.67–1.00)	0.052	0.94 (0.74–1.18)	0.57
**Presence of children in the household**^**#**^												
None	537 (19.9)	[18.4–21.4]	337 (62.8)	[58.71–66.89]	200 (37.2)	[33.11–41.29]	1.90	0.17	Ref.		Ref.	
Yes, at least one	2,168 (80.1)	[78.6–81.6]	1,290 (59.5)	[57.43–61.57]	878 (40.5)	[38.43–42.57]			0.87 (0.72–1.06)	0.17	0.89 (0.72–1.10)	0.29
**Perceived health status**												
Good-excellent	2,650 (98.0)	[97.47–98.53]	1,592 (60.1)	[58.24–61.96]	1,058 (39.9)	[38.04–41.76]	0.285	0.59	Ref.		Ref.	
Poor- very poor	55 (2.0)	[1.47–2.53]	35 (63.6)	[50.88–76.32]	20 (36.4)	[23.68–49.12]			1.16 (0.67–2.03)	0.59	1.14 (0.65–2.08)	0.65
**Vaccination experience and perception**												
**Flu vaccine in the last 2 years**												
Yes, at least once	727 (26.9)	[25.23–28.57]	547 (75.2)	[72.06–78.34]	180 (24.8)	[21.66–27.94]	94.48	^****^	Ref.		Ref.	
Never	1,978 (73.1)	[71.43–74.77]	1,080 (54.6)	[52.41–56.79]	898 (45.4)	[43.21–47.59]			0.40	^****^	0.36	^****^
									(0.33–0.48)		(0.30–0.44)	
**Vaccine will curb spread of infection**												
Yes	2,079 (76.9)	[75.31–78.49]	1,549 (74.5)	[72.63–76.37]	530 (25.5)	[23.63–27.37]	772.7	^****^	Ref.		Ref.	
No	626 (23.1)	[21.51–24.69]	78 (12.5)	[9.91–15.09]	548 (87.5)	[84.91–90.09]			0.05 (0.04–0.06)	^****^	0.05 (0.04–0.07)	^****^
**COVID-19 experience and perception**												
**Infected with COVID-19**												
Yes	95 (3.5)	[2.81–4.19]	61 (64.2)	[54.56–73.84]	34 (35.8)	[26.16–45.44]	0.678	0.41	Ref.		Ref.	
No	2,610 (96.5)	[95.81–97.19]	1,566 (60.0)	[58.12–61.88]	1,044 (40.0)	[38.12–41.88]			0.84 (0.55–1.28)	0.41	0.93 (0.59–1.44)	0.74
**Know someone infected with COVID-19**												
Yes	2,172 (80.3)	[78.8–81.8]	1,324 (61.0)	[58.95–63.05]	848 (39.0)	[36.95–41.05]	3.02	0.08	Ref.		Ref.	
No	533 (19.7)	[18.2–21.2]	303 (56.8)	[52.59–61.01]	230 (43.2)	[38.99–47.41]			0.84 (0.70–1.02)	0.08	0.86 (0.71–1.06)	0.16
**Perceived personal risk of contracting COVID**–**19**												
Low- very low	1,064 (39.3)	[37.46–41.14]	593 (55.7)	[52.72–58.68]	471 (44.3)	[41.32–47.28]	20.90	^****^	Ref.		Ref.	
Medium	1,145 (42.3)	[40.44–44.16]	698 (61.0)	[58.18–63.82]	447 (39.0)	[36.18–41.82]			1.24 (1.05–1.47)	^*^	1.22 (1.02–1.46)	^*^
High- very high	496 (18.3)	[16.84–19.76]	336 (67.7)	[63.58–71.82]	160 (32.3)	[28.18–36.42]			1.67 (1.33–2.09)	^****^	1.71 (1.35–2.17)	^****^
**Perceived average UAE public risk of contracting COVID-19**												
Low- very low	489 (18.1)	[16.65–19.55]	272 (55.6)	[51.2–60.0]	217 (44.4)	[40.0–48.8]	18.40	^***^	Ref.		Ref.	
Medium	1,367 (50.5)	[48.62–52.38]	795 (58.2)	[55.59–60.81]	572 (41.8)	[39.19–44.41]			1.11 (0.90–1.37)	0.33	1.27 (1.01–1.58)	^*^
High- very high	849 (31.4)	[29.65–33.15]	560 (66.0)	[62.81–69.19]	289 (34.0)	[30.81–37.19]			1.55 (1.23–1.94)	^***^	1.84 (1.44–2.36)	^****^
**Expected effect of COVID-19 on own health**												
Minimal	2,383 (88.1)	[86.88–89.32]	1,390 (58.3)	[56.32–60.28]	993 (41.7)	[39.72–43.68]	27.60	^****^	Ref.		Ref.	
High effect	322 (11.9)	[10.68–13.12]	237 (73.6)	[68.86–78.34]	85 (26.4)	[21.66–31.14]			1.99 (1.53–2.59)	^****^	2.05 (1.57–2.70)	^****^
**Current COVID-19 situation is serious**												
Agree	1,880 (69.5)	[67.77–71.23]	1,282 (68.2)	[66.1–70.3]	598 (31.8)	[29.7–33.9]	166.4	^****^	Ref.		Ref.	
Disagree	825 (30.5)	[28.77–32.23]	345 (41.8)	[38.43–45.17]	480 (58.2)	[54.83–61.57]			0.34 (0.28–0.40)	^****^	0.35 (0.29–0.42)	^****^
**Not understanding what is happening in this COVID-19 pandemic**												
Agree	989 (36.6)	[34.78–38.42]	581 (58.7)	[55.63–61.77]	408 (41.3)	[38.23–44.37]	1.28	0.26	Ref.		Ref.	
Disagree	1,716 (63.4)	[61.58–65.22]	1,046 (61.0)	[58.69–63.31]	670 (39.0)	[36.69–41.31]			1.1 (0.94–1.29)	0.26	1.09 (0.92–1.29)	0.33
**Authorities are doing good job in handling COVID-19 pandemic**												
Agree	2,539 (93.9)	[93.0–94.8]	1,543 (60.8)	[58.9–62.7]	996 (39.2)	[37.3–41.1]	6.72	^**^	Ref.		Ref.	
Disagree	166 (6.1)	[5.2–7.0]	84 (50.6)	[42.99–58.21]	82 (49.4)	[41.79–57.01]			0.66 (0.48–0.91)	^**^	0.63 (0.45–0.88)	^**^

*aOR, adjusted odds ratio; CI, confidence interval; COR, crude odds ratio*.

a *Percentages presented as per total of the variable rather than the grand total of the study sample*.

# *Covariates from the sociodemographic group introduced into the logistic regression as the univariate analysis with Chi Square testing had a p-value <0.25*.

* *p <0.05; *

** *p < 0.01;*

*** *p < 0.001;*

**** *p < 0.0001*.

### COVID-19 and Vaccination Perceptions

Over two-thirds (73.1%) of participants stated not receiving the influenza vaccination within the past 2 years; additionally, over two-thirds (76.9%) believed that the COVID-19 vaccination would help curb the spread of the infection and aid in controlling the pandemic.

There were 95 (3.5%) participants who stated having been infected by COVID-19, and the majority of the participants (80.3%) reported knowing someone who has contracted the disease. Nearly two-thirds (66.6%) of participants perceived their own personal risk of contracting the disease being medium-very high, while 81.9% perceived the general UAE public risk to be medium-very high. Most participants (88.1%) stated the expected effect of COVID-19 on their own health if they contracted it to be minimal. Nearly one-third (30.5%) of the participants did not believe that the current COVID-19 situation is serious. Moreover, over one-third (36.6%) expressed that they do not understand what is happening in the current COVID-19 pandemic. However, 93.9% of the participants believed that the authorities are doing a good job in handling it. A further summary of the COVID-19 perception is summarized in Table 1.

### Willingness for the Future COVID-19 Vaccination

Out of the 2,705 participants, 1,627 (60.1%) expressed their willingness to take the COVID-19 vaccination in the future if proven effective. To explore the association between this willingness and the various factors in the questionnaire, multivariable logistic regression analysis was carried out and adjusted for all of the sociodemographic factors except for the perceived health status as it did not meet the criteria of introduction for a *p*-value <0.25 in the univariate chi-square analysis (summary of the univariate analysis is provided in Table 1).

Male participants were more likely to express their willingness to take the COVID-19 vaccine than females (aOR 2.37, 95% CI 1.92–2.93, *p* < 0.0001). Similarly, non-Emiratis were more willing to take the vaccine (aOR 1.45, 95% CI 1.20–1.76, *p* < 0.001). However, when looking at the age groups, older participants were less willing to take the vaccine than the 18–24 age group, with people aged 55 or older having a 0.32 adjusted odds ratio (95% CI 0.19–0.51, *p* < 0.0001). Compared to Dubai, Abu Dhabi residents did not show a difference in their willingness to take the vaccine; however, residents of Sharjah and the Northern Emirates showed a statistically significant higher willingness to take the vaccine in the future (aOR 1.26, 95% CI 1.02–1.56, *p* < 0.05; aOR 1.88, 95% CI 1.43–2.49, *p* < 0.0001, respectively). Likewise, compared to postgraduates, participants who had an undergraduate degree or less were more willing to take the vaccine, as summarized in Table 1. On the other hand, students did not show any statistically significant difference in their willingness compared to the employed group. However, unemployed individuals, including retired, were less willing to take the vaccine (aOR 0.72, 95% CI 0.60–0.88, *p* < 0.001). There was no statistical difference in the willingness for vaccination based on the number of adults in the household, the presence of children in the household, or the perceived health status, as summarized in Table 1.

Individuals that did not receive the influenza vaccine within the last 2 years and the ones that did not believe that the vaccine would help in curbing the spread of the COVID-19 infection were less likely to report willingness to take the COVID-19 vaccination (aOR 0.36, 95% CI 0.30–0.44, *p* < 0.0001; aOR 0.05, 95% CI 0.04–0.07, *p* < 0.0001, respectively). Furthermore, there was no statistically significant association between having been infected by COVID-19 or knowing someone who had and the willingness to take the vaccine. On the other hand, participants that perceived either their own risk or the general public's risk of contracting the infection as being high-very high showed a higher willingness to take the vaccine (aOR 1.71, 95% CI 1.35–2.17, *p* < 0.0001; aOR 1.84, 95% CI 1.44–2.36, *p* < 0.0001, respectively). Likewise, perceiving the effect of the disease to have a higher effect on one's personal health showed higher odds for vaccination (aOR 2.05, 95% CI 1.57–2.70, *p* < 0.0001). There was no statistically significant association between perceived understanding of the pandemic situation and the willingness for vaccination (see Table 1). Lastly, individuals that did not perceive the COVID-19 situation as being serious and the ones that did not believe that the authorities are handling the pandemic appropriately were less willing to take a COVID-19 vaccination in the future (aOR 0.35, 95% CI 0.29–0.42, *p* < 0.0001; aOR 0.63, 95% CI 0.45–0.88, *p* < 0.01, respectively).

### Stated Reasons for Wanting or Not Wanting to Receive the COVID-19 Vaccination

There were a total of 1,627 individuals willing to receive the COVID-19 vaccination in the future. The top stated reasons were to protect oneself (78.2%) and one's close relatives (73.0%). This was followed by vaccination being a civic duty, recommended by the public authorities, and vaccines being safe, with percentages ranging from 33.6–38.7%. Vaccines not having side effects or being advised by a health professional to receive it were lower in the list (17.5 and 5.9%, respectively). A further summary of the data is provided in Table 2. Of note, the participants were given the opportunity for written comments, and out of 55 comments from individuals willing for vaccination, there were 10 comments stating the reason being to regain normal social life and easiness in travel (data not shown).

**Table 2:** The main stated reasons for wanting or not wanting to receive COVID-19 vaccination in the future (*n* = 2705).

	***N***	**%**	**[95% CI]**
**Main reasons for wanting to receive the vaccine:** [can choose more than one]	**1,627**	**60.1**	**[58.25–61.95]**
Protecting myself	1,273	78.2	[76.19–80.21]
Protecting my close relatives	1,187	73.0	[70.84–75.16]
A health professional advised me to get vaccinated	96	5.9	[4.76–7.04]
Getting vaccinated is a civic duty	629	38.7	[36.33–41.07]
Vaccination is recommended by public authorities	546	33.6	[31.31–35.89]
Vaccines are safe	602	37.0	[34.65–39.35]
Vaccines have no side effects	285	17.5	[15.65–19.35]
Other	42	2.6	[1.83–3.37]
**Main reasons for not wanting to receive the vaccine:** [can choose more than one]	**1,078**	**39.9**	**[38.05–41.75]**
Vaccines are not safe enough	608	56.4	[53.44–59.36]
Vaccines have side effects	558	51.8	[48.82–54.78]
Novel coronavirus (COVID-19) is not a severe disease	222	20.6	[18.19–23.01]
Vaccines lack efficacy	148	13.7	[11.65–15.75]
A health professional advised me to avoid vaccination	95	8.8	[7.11–10.49]
I don't think I will catch the disease	21	1.9	[1.09–2.71]
I have medical reasons to avoid the vaccine	60	5.6	[4.23–6.97]
Only people with medical problems should be vaccinated	151	14.0	[11.93–16.07]
People should develop immunity naturally rather than through a vaccine	453	42.0	[39.05–44.95]
Other	153	14.2	[12.12–16.28]

*Percentages do not add up to 100 as they are per option, and the participants are allowed to select more than one option*.

*CI, confidence interval*.

On the other hand, there were 1,078 individuals not willing to receive the COVID-19 vaccine in the future. The top two reasons were that the vaccines are not safe enough (56.4%) and have side effects (51.8%). This was followed by the belief that people should develop immunity naturally rather than through a vaccine (42.0%). Further, 20.6% of individuals believed that COVID-19 is not a severe disease that warrants vaccination. Some of these participants also believed that only individuals with medical problems (14.0%) should be vaccinated, and 13.7% believed that the vaccines lack efficacy. Lower in the list were having a medical reason not to be vaccinated, a health professional advising the person to avoid the vaccine, and a belief that the person will not contract the disease, with each of these reasons stated <10% of the time (see Table 2). Of note, there were a total of 150 written comments; 75 of them stated the reason being that the clinical trials are too rushed and of short duration such that long-term safety cannot be proven, and that at least 1–2 years are needed to provide a clearer safety profile (data not shown). Some also added that in such a situation, if they received the vaccine and some complications happen in the future, there will be no compensation scheme as they were received out of a clinical trial setting. Additionally, we received five comments of participants concerned about safety in pregnancy and thus not willing to take the vaccine.

## Discussion

The current study has shown that 60.1% of participants were willing to take the COVID-19 vaccination in the future. This finding echoes some similarities to various studies and polls conducted worldwide (11–13). For instance, one study in Italy showed vaccine acceptance at 59% level, and another study in the USA showed acceptance at 67% level (12, 13). However, most of these studies were conducted earlier in the pandemic, and more recent studies have shown some decline in public acceptance over time. For example, a study conducted by the Pew Research Center in the USA has shown that public acceptance had dropped from 72% in May to 51% in September 2020 (17). Consequently, it is of paramount importance to track such changes over time and study the reasons behind the changes in public opinion in order to maximize their acceptance of the vaccine once it becomes available. There are various platforms of vaccine being studied, and some of them quite novel; thus, some hesitancy might stem from the novelty of the platforms and the associated unknowns. Additionally, these vaccines come from various countries, and skepticism about one country or another might influence the acceptance rate for a specific vaccine. Therefore, further studies would be needed to elucidate these potential reasons for hesitancy and how they change over time.

Sociodemographic factors such as being male, non-Emirati, and having a lesser level of education were associated with higher odds for wanting to receive the vaccine. Regarding male gender, once more, there are multiple studies that have shown the male gender to have a higher acceptance rate for the vaccine than females (13, 17–19). This is not a new finding, as previous studies on H1N1 have shown similar findings (14). We speculate that part of the relative hesitancy in the female population is due to circumstance-specific safety issues, such as safety in pregnancy. In our study, we received five different written comments from participants not willing to receive the vaccine, reporting the reason as safety during pregnancy. We also speculate that the effect on fertility might be another worry; however, this and other reasons need to be further studied and delineated. Vaccine studies, whether in the earlier phases or the post marketing phase, need to address and study the issue of fertility and pregnancy to gain the trust and support of this important segment of society.

Pertaining to the educational level of participants, contrary to most studies, our study has shown that the higher the participant's level of education, the greater the hesitancy toward receiving the vaccine (13, 14, 17). One possible explanation for this is that with a higher level of education, people are more aware of potential side effects and risks associated with newer vaccines, and thus, they become more hesitant. Again, this needs to be further studied at the local and regional level as most of the studies discussed earlier were conducted in Western countries with different cultural and educational backgrounds. Therefore, a different, more targeted regional approach might need to be explored. Similarly, older age groups and unemployed individuals were less likely to accept the vaccine in the future. The higher acceptance rate amongst the younger age group in a country with a small proportion of elderly population and a low death rate compared to other parts of the world might advise a different vaccine distribution strategy. While the vaccination campaign might start by addressing the frontline staff and the elderly, it can be swiftly moved into the younger population that is more accepting of the vaccine in order to create an earlier herd immunity bubble to protect the hesitant older population. The younger population might, in turn, encourage their elders to take the vaccine once they observe its safety in the younger generations.

Participants' past behavior toward vaccinations seems to predict their current vaccination willingness to some degree, as the ones who did not receive the influenza vaccine within the past 2 years were more hesitant to accept the COVID-19 vaccine in the future. This finding is in keeping with other similar studies conducted in different parts of the world (13, 17). Therefore, approaches to overcome influenza vaccine hesitancy might prove to be effective in overcoming the future COVID-19 vaccination hesitancy with clear and transparent campaigns about the vaccination importance, safety profile, and any short or long term side effects, whether found in clinical trials or discovered in the post-marketing phase (20).

Furthermore, participants' beliefs such as not thinking that the vaccine will curb the spread of infection, not thinking of the COVID-19 situation as serious, and not thinking that the authorities are doing a good job in handling the situation showed greater hesitancy in accepting the vaccine in the future. On the other hand, participants' perception about the increased likelihood of contracting the disease by themselves or by the general public and that its effect on their own health will be marked showed a greater association with the willingness to take the vaccine. This is in keeping with similar studies conducted in Indonesia and China (18, 21). Consequently, this might be a useful message to consolidate the acceptance for the vaccine in this group during the COVID-19 vaccination campaign (20, 22). With about 94% of participants approving the government response to the pandemic, this might provide a crucial insight into a successful vaccination campaign. For instance, government and public figures can lead by example and be the first to receive the vaccine once available to encourage the rest of the population. Moreover, official mandates can be made, such as requiring people working for the government and public sector to receive the vaccine, travelers into and out of the country to be required to take it, as well as newly employed immigrants. Furthermore, transparency with the general public regarding the vaccine efficacy and any potential adverse effects will help strengthen trust in the government. All areas of concern need to be evaluated and addressed as the vaccine is being rolled out.

To our surprise, there were no increased odds for wanting to take the vaccine based on the participants' experience with COVID-19 as a disease, either by contracting it or knowing someone who had. This adds to the evidence that vaccine hesitancy is more due to personal beliefs and perceptions of the individuals rather than the factual situation on the ground (23). Furthermore, it highlights that the hesitancy concerns the vaccine itself and its potential feared harms rather than the worry about the disease itself, as will be discussed next. Another potential explanation could be the low infection and death rates experienced in the country at the time the study was conducted, and therefore, some participants might not have seen the urgency of the situation compared to other nations worldwide.

Top stated vaccine hesitancy reasons were vaccine safety and side effects, especially in the long-term; this was further emphasized by the participants' written comments. This is in keeping with various other studies, such as the one by the Pew Research Center and a European study conducted by Neumann-Bohme and colleagues (17, 19, 24). While the medical and scientific communities have been racing to develop a safe and effective vaccine since the start of the pandemic, this seems to generate a major cause of concern due to the short duration of the clinical trials, and some authorities worldwide rushing into authorizing or promising earlier authorization of the vaccine prior to the full completion of the clinical trials (9, 19, 25). Once more, if one is to conduct a successful vaccination campaign, a clear and transparent process needs to be in place to gain public trust throughout the rolling out process of the vaccine.

The anti-vaccine movement, conspiracy theorists, and the belief that one needs to develop immunity naturally rather than through a vaccine are likely to be some of the major hurdles to the future COVID-19 vaccination program (20, 23, 25, 26). In our study, 42% of the participants who were not willing for the vaccine stated the need to develop immunity naturally rather than through a vaccine as one of their top reasons. Therefore, considerable coordinated effort needs to take place locally and globally to challenge such beliefs, especially in the era of social media, globalization, and the rapid spread of information worldwide (20, 22, 27, 28).

It is worth noting that the above discussion was written prior to the UAE starting its vaccination campaign. However, during the review process of the manuscript, the UAE has launched its vaccination campaign by the end of 2020. As of the end of June 2021, over 90% of the UAE adult population has received at least one dose of a COVID-19 vaccine, which is higher than the expected rate based on the current study (29). We attribute this partly to the increased trust in the vaccination that developed as evidence of efficacy and safety evolved, in addition to various successful strategies used by the government to encourage and facilitate the vaccination process for the target population.

## Study Limitations

This was a questionnaire-based cross-sectional survey, thus suffers from the inherent weaknesses of this type of studies, such as recall bias. Additionally, there is a weakness in the study sample selection via the snowballing technique resulting in some selection bias and non-homogenous sampling. While the ideal situation asks for a randomized sample that is representative of the whole country, this proved very challenging on multiple fronts. Firstly, the country is composed of multiple nationalities with limited official statistics about their proportions apart from few unofficial accounts. Secondly, there is no available database of a representative population that one can withdraw the study sample from similar to the databases available in more advanced countries. Lastly, at a time of pandemic with restricted movements and rapidly advancing situation and development of a vaccine, to overcome these challenges and produce a randomized sample representative of the whole population proved extremely challenging. Consequently, we opted for a less rigorous sampling technique despite all the drawbacks associated with it. Having some insight into the vaccine hesitancy and then building the investigation further afterward is better than having no information at all. Therefore, the study results reflect the outcome of the study sample and cannot be generalized to the whole UAE general population.

The study was provided in Arabic and English only; therefore, the study only reflects the views of individuals who are literate in either of these languages. Moreover, the questionnaire was only circulated via the WhatsApp messaging platform; consequently, participation in the study was limited to the individuals with access to this platform. Additionally, limiting the participants' answers to a binary option of willing or not willing to take the vaccine does not capture the breadth of the responses, as some participants might not be decided, while others might be strongly or somewhat likely to opt for or against the vaccination.

## Conclusion

This study adds to the growing body of evidence worldwide that COVID-19 vaccination hesitancy is significantly high among different populations. Here in the UAE, it was as high as nearly 40% in our study. This finding needs to be followed up with a more representative sampling of the whole population study to confirm the findings. In the current study, being male and of younger age were associated with greater vaccine acceptance. This, if proved true in the general population, might ask for a different vaccination strategy in which vaccination starts at the most vulnerable groups and then swiftly shifted to the younger population to create a bubble of herd immunity and encourage the hesitant elderly to take it thereafter. Similarly, a greater perception of risk of contracting the infection and perception that it might have a significant effect on one's health significantly increased the odds of wanting to receive the vaccine. This asks for seizing the moment in the vaccination campaign. Suppose the pandemic situation gets worse, then emphasizing the seriousness of the situation and the importance of the vaccine might gain a wider acceptance compared to a situation that has lower infection and death rates. Over 50% of participants who are vaccine-hesitant stated their main worry being vaccine safety and presence of side effects, especially in the long-term, which cannot be ascertained in the short duration of the current vaccine studies that are being carried out worldwide. Additionally, the belief that one needs to develop immunity naturally rather than through vaccination was one of the highest reasons. Consequently, it is predicted that once the COVID-19 vaccine becomes available, such challenges will arise. A successful COVID-19 campaign would require a significant preparation to overcome these hurdles, misinformation, and worries through a coordinated global effort and campaign in the era of vast social media and rapid COVID-19 infodemic. Gaining public trust through a continuous, transparent, and updated campaign regarding the importance of the vaccine for the individual and the community locally and globally, and the expected short- and long-term side effects would be of utmost importance.

## Data Availability Statement

The original contributions presented in the study are included in the article/Supplementary Material, further inquiries can be directed to the corresponding author/s.

## Ethics Statement

The studies involving human participants were reviewed and approved by Dubai Scientific Research Ethics Committee at Dubai Health Authority. The patients/participants provided their written informed consent to participate in this study.

## Author Contributions

ShahA transferred the questionnaire into an online form. AHA was responsible for data analysis and writing the draft of the paper. All authors contributed to the design of the study, data collection, review of the draft manuscript and subsequent modifications. All authors reviewed the manuscript before submission and approved the final version.

## Conflict of Interest

The authors declare that the research was conducted in the absence of any commercial or financial relationships that could be construed as a potential conflict of interest.

## Publisher's Note

All claims expressed in this article are solely those of the authors and do not necessarily represent those of their affiliated organizations, or those of the publisher, the editors and the reviewers. Any product that may be evaluated in this article, or claim that may be made by its manufacturer, is not guaranteed or endorsed by the publisher.

## Abbreviations

aOR: adjusted odds ratio
CI: confidence interval
COVID-19: coronavirus disease 2019
SARS-CoV-2: severe acute respiratory syndrome-coronavirus-2
UAE: United Arab Emirates.
