# Supplementary material for: COVID-19 Vaccine Acceptance in a Sample From the United Arab Emirates General Adult Population: A Cross-Sectional Survey, 2020
Source: Front Public Health. 2021 Jul 26;9:614499. doi: 10.3389/fpubh.2021.614499 (PMC8350048; doi:10.3389/fpubh.2021.614499)
Supplement: Supplementary file 1 [file Data_Sheet_1.PDF]

Public Perception Towards COVID-19 Vaccination among UAE Population in 2020, A Cross-Sectional Survey

This study aims at evaluating your perception and attitude towards the future COVID-19 vaccination. It comprises a total of 23 questions. Try answering them to the best of your knowledge. Your participation in this survey is anonymous and all information will be kept confidential.

Demographics

For each of the following questions tick one answer that applies to you:

1. **Please state your current place of residence:**
   - UAE
   - Outside UAE

[If a participant selects outside UAE, the survey will take him to a thank you page stating that the survey is currently only available for people residing in UAE]

2. **Which emirate do you currently reside in:**
   - Abu Dhabi
   - Dubai
   - Sharjah
   - Ajman
   - Ras al-Khaimah
   - Umm al-Quwain
   - Fujairah

3. **Gender:**
   - Male
   - Female

4. **Age:**
   - Under 18
   - 18-24

- 25-34
- 35-44
- 45-54
- 55-64
- 65+

[If a participant selects under 18, the survey will take him to a thank you page stating that the survey is currently only available for people 18 years of age and older]

5. Nationality:

- UAE national
- Arab
- Other non-Arab

6. Your highest educational qualification:

- No schooling
- Less than high school
- Completed high school
- University undergraduate level
- Postgraduate level

7. Your current work status:

- Employed (including self-employed and retired self-employed)
- Unemployed (including retired)
- Student

8. Number of adults in the household:

- One
- Two
- More than two

9. Number of children under 18 years of age in the household:

- None
- One
- More than one

10. How do you perceive your current health status:

- Very poor
- Poor
- Good
- Very good
- Excellent

Vaccination willingness and experience

11. Have you taken the seasonal flu vaccine in the last two years:

- Never
- Yes, at least once

12. If the novel coronavirus (COVID-19) vaccine becomes available and proven to be effective, are you willing to take it:

- Yes [go to question 13a]
- No [go to question 13b]
- I have already received it as part of a clinical trial [skip to question 14]

13a. Your main reason(s) for wanting to receive the vaccine: (tick all that applies)

- ○ Protecting myself
- ○ Protecting my close relatives
- ○ A health professional advised me to get vaccinated
- ○ Getting vaccinated is a civic duty
- ○ Vaccination is recommended by public authorities
- ○ Vaccines are safe
- ○ Vaccines have no side effects
- ○ Other (specify):

13b. Your main reason(s) for not wanting to receive the vaccine: (tick all that applies)

- ○ Vaccines are not safe enough
- ○ Vaccines have side effects
- ○ Novel coronavirus (COVID-19) is not a severe disease
- ○ Vaccines lack efficacy
- ○ A health professional advised me to avoid vaccination
- ○ I don't think I will catch the disease
- ○ I have medical reasons to avoid the vaccine
- ○ Only people with medical problems should be vaccinated
- ○ People should develop immunity naturally rather than through a vaccine

- Other (specify):

14. Do you think that the COVID-19 vaccine will help in eradicating or controlling the spread of infection:

- Yes
- No

COVID-19 perception

15. Did you get infected by the COVID-19 coronavirus:

- Yes
- No

16. Do you know someone who has contracted the COVID-19 coronavirus infection:

- No
- Yes, a family or a friend
- Yes, but not a very close person to me

17. What level of risk do you think the average UAE resident has of catching the novel coronavirus (COVID-19) infection during this pandemic?

- Very High
- High
- Medium
- Low
- Very low

18. What level of risk do you think you have of catching the novel coronavirus (COVID-19) infection during this pandemic?

- Very High
- High
- Medium
- Low
- Very low

19. If you were infected with the novel coronavirus (COVID-19), how seriously do you think it would affect your health?

- Not at all

- Somewhat affect
- Very seriously affect
- Extremely

20. I think the current novel coronavirus (COVID-19) infection situation is serious

- Agree
- Disagree

21. I do not understand what is happening with this novel coronavirus (COVID-19) pandemic

- Agree
- Disagree

22. In general, I think the authorities are doing a good job of dealing with the novel coronavirus (COVID-19) infection pandemic

- Agree
- Disagree

الإدراك والفهم العام لمجتمع الإمارات نحو تطعيم كوفيد-19 في سنة 2020، دراسة مسح مقطعية

تهدف هذه الدراسة إلى تقييم نظرتك وانطباعك نحو تطعيم كوفيد-19 المستقبلي، تشتمل الدراسة على 23 سؤالا، حاول الإجابة عليهم حسب علمك على أحسن وجه. إن مشاركتك في هذا المسح ستتم بصورة مجهولة الهوية كما سيتم المحافظة على جميع المعلومات بصورة سرية.

البيانات الشخصية

1. يرجى تحديد مكان إقامتك الحالية:

- داخل دولة الإمارات العربية المتحدة
- خارج دولة الإمارات العربية المتحدة

2. في أي إمارة تقيم حاليًا:

- أبو ظبي
- دبي
- الشارقة
- عجمان
- رأس الخيمة
- أم القيوين
- الفجيرة

3. الجنس:

- ذكر
- أنثى

4. ما هو عمرك:

- أقل من 18
- 24-18
- 34-25
- 44-35

- 54-45
- 64-55
- +65

5. الجنسية:

- إماراتي
- عربي
- أجنبي غير عربي

6. أعلى مستوى تعليمي أنجزته:

- غير متعلم
- أقل من التعليم المدرسي الثانوي
- أكملت التعليم المدرسي الثانوي
- مستوى جامعي- بكالوريوس
- مستوى جامعي- ما بعد البكالوريوس

7. وضعك الوظيفي الحالي:

- موظف (يشمل ذلك العاملين لحسابهم الخاص والمتقاعدين العاملين لحسابهم الخاص)
- عاطل عن العمل (يشمل ذلك المتقاعد)
- طالب

8. عدد الأفراد البالغين الساكنين في المنزل:

- واحد
- اثنان
- أكثر من اثنين

9. عدد الأطفال ما دون سن الثامنة عشر (18) الساكنين في المنزل:

- لا يوجد
- واحد
- أكثر من واحد

10. كيف تقيم حالتك الصحية الحالية؟

- ضعيفة جدا
- ضعيفة
- جيدة
- جيدة جدا
- ممتازة

الرغبة في التطعيم والخبرة السابقة

11. هل سبق وأن أخذت لقاح الأنفلونزا الموسمية في العامين الماضيين؟

- لا
- نعم، مرة واحدة على الأقل

12. إذا أصبح لقاح فيروس كورونا المستجد (كوفيد-19) متوفرًا وثبت فعاليته، هل أنت على استعداد لأخذه:

- نعم [انتقل إلى السؤال 13 أ]
- لا [انتقل إلى السؤال 13 ب]
- لقد تلقيته بالفعل كجزء من تجربة إكلينيكية [انتقل إلى السؤال 14]

13 أ .السبب (الأسباب) الرئيسية لرغبتك في تلقي التطعيم: (اختر كل ما ينطبق)

- لحماية نفسي
- لحماية أقاربي
- نصحني أحد المتخصصين الصحيين بالتطعيم
- التطعيم واجب مدني
- توصي السلطات العامة بالتطعيم
- التطعيم آمن
- ليس للتطعيمات أي آثار جانبية
- أسباب أخرى (حدد):

13 ب .السبب (الأسباب) الرئيسية لعدم رغبتك في تلقي التطعيم: (اختر كل ما ينطبق)

- اللقاحات ليست آمنة بما يكفي
- اللقاحات لها آثار جانبية
- لا يعد فيروس كورونا المستجد (كوفيد-19) مرضًا خطيرًا
- تفتقر اللقاحات إلى الفعالية
- نصحني أحد الأخصائيين الصحيين بتجنب التطعيم
- لا أعتقد أنني سأصاب بالمرض
- لدي أسباب طبية لتجنّب التطعيم
- فقط الأشخاص الذين يعانون من مشاكل طبية يجب تطعيمهم
- يجب اكتساب المناعة بشكل طبيعي وليس من خلال اللقاح
- أسباب أخرى (حدد):

14. هل تعتقد أن لقاح فيروس كورونا المستجد (كوفيد-19) سيساعد في القضاء على العدوى أو الحد من انتشارها؟

- نعم

- لا

التصور لمرض فيروس كورونا المستجد (كوفيد-19)

15. هل سبق وأن أصبت بفيروس كورونا المستجد (كوفيد-19):

- نعم
- لا

16. هل تعرف شخصًا أصيب بفيروس كورونا المستجد (كوفيد-19):

- لا
- نعم، أحد أفراد العائلة أو صديق
- نعم، ولكن ليس شخصا مقربا مني

17. من وجهة نظرك، ما هي احتمالية إصابة الفرد القاطن في دولة الإمارات العربية المتحدة بفيروس كورونا المستجد (كوفيد-19) خلال فترة هذه الجائحة؟

- مرتفعة جدًا
- مرتفعة
- متوسطة
- منخفضة
- منخفضة جدًا

18. من وجهة نظرك، ما هي احتمالية إصابتك بفيروس كورونا المستجد (كوفيد-19) خلال فترة هذه الجائحة؟

- مرتفعة جدًا
- مرتفعة
- متوسطة
- منخفضة
- منخفضة جدًا

19. في رأيك إذا أصبت بفيروس كورونا المستجد (كوفيد-19) ماذا ستكون درجة خطورة تأثيره على صحتك؟

- لا أعتقد أن لديه أي تأثير على صحتي
- مؤثر إلى حد ما
- مؤثر بدرجة خطرة جدًا
- تأثير شديد للغاية

20. أعتقد أن الوضع الحالي لعدوى فيروس كورونا المستجد (كوفيد-19) خطير وجدي:

- أتفق مع هذه العبارة
- لا أتفق

21. أشعر أني لا أدرك ما الذي يحدث في جائحة فيروس كورونا المستجد (كوفيد-19):

- أتفق مع هذه العبارة
- لا أتفق

22. بشكل عام، أعتقد أن السلطات تقوم بعمل جيد في التعامل مع جائحة فيروس كورونا المستجد (كوفيد-19):

- أتفق مع هذه العبارة
- لا أتفق
